# Flow Cytometric Detection of the Classical Hodgkin Lymphoma: Clinical and Research Applications

**DOI:** 10.1155/2011/387034

**Published:** 2010-11-28

**Authors:** Mikhail Roshal, Brent L. Wood, Jonathan R. Fromm

**Affiliations:** ^1^Department of Laboratory Medicine, University of Washington, Seattle, WA 98195, USA; ^2^Department of Pathology and Laboratory Medicine, Weill Cornell Medical College, New York, NY 10065, USA

## Abstract

Classical Hodgkin lymphoma (CHL) is a relatively uncommon B cell-derived neoplasm that presents with rare malignant cells in an abundant reactive background. The diagnosis of CHL currently relies on a combination of morphologic findings and immunohistochemical stains. With the exception of rare cases with dramatically increased malignant populations, isolation of pure viable tumor cells has not been historically possible. Recently, a reliable flow cytometric assay for direct detection and isolation of the malignant cells in this disease has been developed. This assay has proven useful diagnostically and has been clinically validated to have a very high sensitivity and nearly absolute specificity for the diagnosis of CHL in routine clinical samples. This paper describes the methodology for the flow cytometric detection of CHL in clinical samples as well as current state of evaluation of background lymphocytes as an adjunct diagnostic test. Also discussed are exciting research applications of the direct isolation of viable tumor cells in CHL. The current state of flow cytometric evaluation of nodular lymphocyte predominant Hodgkin lymphoma and T cell-rich large B cell lymphoma is also briefly discussed.

## 1. Clinical Diagnosis of Classical Hodgkin Lymphoma by Flow Cytometry

With increasingly rare exceptions, diagnosis of lymphoproliferative diseases and myeloid stem cell disorders relies on a multimodal analysis in which flow cytometry plays a significant role [1]. While a detailed technical overview of flow cytometry is beyond the scope of this paper and can be found elsewhere [1], flow cytometry immunophenotyping relies on the detection of individual cells in liquid phase by using antigen expression and their light scatter properties. The antibody-stained cells are characterized by detecting signals from antibody-linked laser-activated fluorescent tags. By using combinations of antibody-linked tags with distinct excitation and/or emission spectra, a single cell can be interrogated for the presence and intensity of expression of multiple antigens. Newer digital acquisition multilaser flow cytometers allow for rapid detection of millions of individual cells with simultaneous evaluation of ten or more antigens in a single analysis.

The relatively rapid analytical time makes flow cytometry an attractive initial diagnostic modality. This analysis can guide further morphologic and molecular workup leading to savings in cost and time of the total evaluation. In many cases, the immunophenotype obtained by flow cytometry alone can be diagnostic for specific hematologic neoplasms in a correct clinical and morphologic context. Because of the ability to analyze a large number of cells, flow cytometry is ideally suited for the detection of relatively rare, antigenically distinct populations within complex cell mixtures. This ability has been successfully exploited clinically for the detection of relatively low levels of minimal residual disease (MRD) posttreatment in leukemia and multiple myeloma is now considered one of the critical modalities to monitor MRD [1–4]. It also appears to be ideally suited for the detection of lymphomas where the malignant population is relatively rare, and most of the cells belong to a benign inflammatory background. Examples of such disorders include classical Hodgkin lymphoma (CHL), nodular lymphocyte predominant Hodgkin lymphoma (NLPHL), and T cell-rich large B cell lymphoma (TCRLBCL).

Classical Hodgkin lymphoma is a B cell neoplasm where the neoplastic population represents less than 1% and frequently less than 0.01% of the total cells [5]. The neoplastic population, referred to as Hodgkin or Reed-Sternberg (HRS) cells, can often be recognized morphologically by large cell size, sometimes multilobated nuclei, and characteristically prominent nucleoli. Until recently, the diagnosis relied exclusively on the morphologic appearance of the tumor in fixed paraffin-embedded tissue sections. The development of the technique to stain cells in tissue sections with antibody (immunohistochemistry) has added significantly to the specificity of the diagnosis and allowed for further tissue-based analysis of the immunophenotype of the HRS cells. Over the past twenty years or so, immunohistochemical studies have uncovered numerous surface antigens for the reliable detection of HRS cells in tissue sections. Among these, expression of CD30 and CD15 combined with lack of expression of CD20 (the B cell antigen), CD45 (pan-hematopoietic antigen), and CD3 (T cell antigen) are used in clinical practice [5]. Additionally, bright CD40 [6] and CD95 [7] expression has been demonstrated in a great majority of CHL cases and could potentially help to distinguish HRS cells from other CD30 positive events in the proper context. Yet, detection of HRS cells by flow cytometry has remained unobtainable.

## 2. Technical Aspect and Remaining Challenges in Clinical Detection of HRS Cells by Flow Cytometry

The inability to detect the HRS cells had often been attributed to their cell lysis during preparations or during the cell acquisition [8]. As later experience has shown, these problems have been overstated. Other significant challenges were indeed present, including the relative rarity of the HRS cells within CHL tumors, rosetting of the neoplastic cells by T cells in cell preparations of CHL biopsies, and the large size of the HRS cells. Recently, a method that allows a routine detection of HRS cells with nearly absolute specificity and high sensitivity for clinical diagnosis of CHL has been developed [9, 10]. This section describes how problems that arose with earlier detection methods have been overcome using newer analytic and cell preparation approaches.

**Figure 1:**
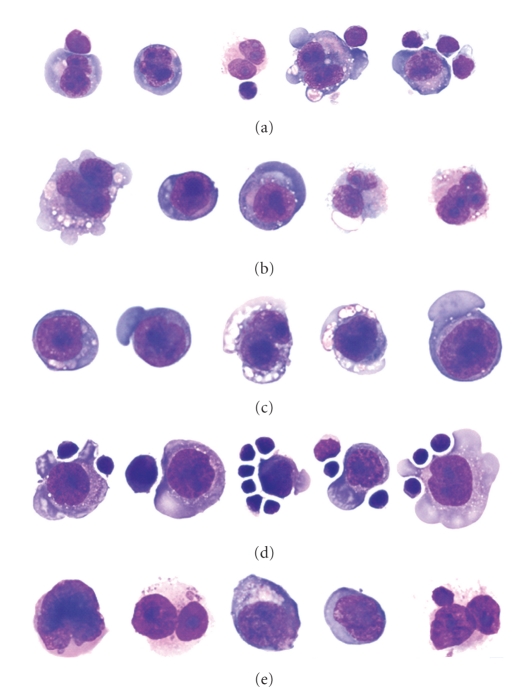
Wright-Giemsa-stained cytomorphology of representative, intact flow cytometric cell-sorted HRS cells from lymph nodes involved by CHL with immunophenotypes consistent with CHL. (a) A CHL case flow sorted in absence of blocking antibodies showing the presence of T cell-HRS cell rosettes. (b) The same case as seen in (a) (above) where flow sorted in the presence of blocking antibodies showing only isolated HRS cells, suggesting the blocking antibodies disrupted the rosettes. (c) A CHL case flow sorted in the presence of blocking antibodies. (d) A CHL case flow sorted in the absence of blocking antibodies showing T cell-HRS cell rosettes. (e) A CHL case flow sorted in the absence of blocking antibodies. (© 2006 American Society of Clinical Pathology [9]).

Normal T cells have been shown to closely associate with HRS cells in immunohistochemical sections and bind (rosette) cell lines derived from CHL tumors. Indeed, using a CHL cell line (L428), Sanders and coworkers have shown that high levels of CD54 (ICAM-1) and CD58 (LFA-3) on the HRS cell bind to LFA-1 and CD2 on the T cell [11]. This phenomenon can be seen on the cytospins of the routine flow cytometric cell suspensions of CHL tumors (see Figure 1). 

**Figure 2:**
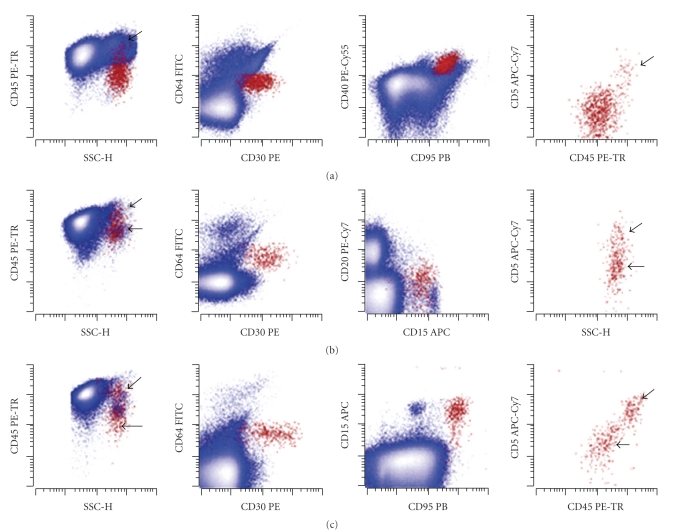
Clinical cases of CHL can demonstrate varying proportions of HRS cells rosetted by CD5+ T cells that also increase the apparent CD45 expression level of the rosetted HRS cells. Representative examples of flow cytometric immunophenotyping of lymph nodes involved by CHL using the single tube, 9-color assay are shown. HRS cells (shown in red and emphasized) are identified by their expression of CD30, CD40, CD95, absence of expression of CD64, and increased side light scatter (SSC-H) compared to normal lymphocytes; all remaining viable events are in blue. Oblique and horizontal arrows denote rosetted and unrosetted HRS populations, respectively. Dot plots at the end of panels demonstrate HRS cells in isolation for clarity. (a) Neoplastic unrosetted HRS cells have expression of low CD45, intermediate CD30, and bright CD40 and CD95, without expression of CD64 (position of negative determined by isotype-matched control experiment, data not shown), CD5, CD15 (data not shown), or CD20 (data not shown). The small, T-rosetted population of HRS has increased CD45 and expression of CD5 (oblique arrow). (b) Neoplastic unrosetted HRS cells have expression of intermediate CD45, intermediate to bright CD30, CD40 (data not shown) and CD95 (data not shown), and intermediate CD15, without expression of CD5, CD64, or CD20. The small rosetted population has expression of CD5 and increased CD45. (c) Neoplastic unrosetted HRS cells have expression of low to intermediate CD45, variable intermediate to bright CD30, bright CD40 (data not shown) and CD95, intermediate CD15, and variable CD71 (data not shown), without expression of CD5, CD64, or CD20 (data not shown). The rosetted population has increased CD45 and expression of CD5. (© Modified from 2009 American Society of Clinical Pathology [10]).

This rosetting causes a significant proportion of HRS cells to fall outside the area that is usually used for recognition of individual (nonaggregated) cells in flow cytometric analysis. The HRS-T cell rosettes fall either in the area that is routinely excluded as coincident events (cell aggregates) or are located at the very edge or beyond the high end of the measurable range of forward scatter parameters set to analyze hematopoietic cells. Moreover, the interaction of HRS cells with the T cells is expected to lead to a composite immunophenotype between these two cell types. Indeed, most cases of CHL cells do demonstrate a composite, HRS-T cell immunophenotype (CD15+, CD30+, CD40+, CD95+, CD20−, CD3+, CD5+, and CD45+). Disrupting interactions between HRS cells and T cells with blocking antibodies reduces rosetting by T cells leading to the expected HRS cell immunophenotype (CD15+, CD30+, CD40+, CD95+, CD20−, CD3−, CD5−, and CD45 dim). In routine clinical practice, blocking antibodies are not necessary with most CHL cases demonstrating both rosetted and unrosetted HRS population. Furthermore, this unique feature of Hodgkin lymphomas can be diagnostically useful (Figure 2).

**Figure 3:**
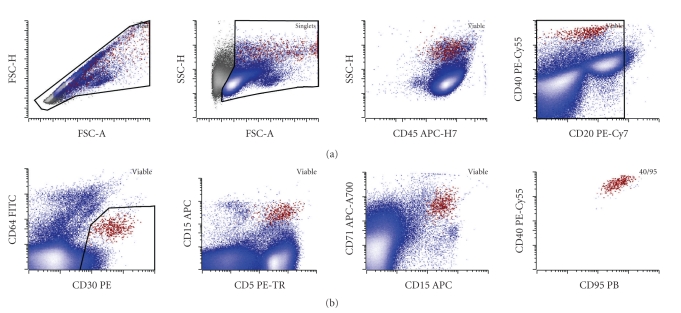
Identification of a small HRS population in a clinical sample: malignant HRS cells shown in red have increased forward scatter area (FSC-A) and height (FSC-H) (note a subpopulation with HRS cells with disproportionately increased FSC-A likely corresponding to a rosetted population) and increased side scatter height (SSC-H) compared to the rest of the node cellularity (blue). The population showed bimodal, but mostly bright, expression of CD45, low levels of a B cell-marker CD20, no significant expression of a monocyte-marker CD64 (mild increase in apparent CD64 expression is due to autofluorescence of the HRS cells, which was previously verified by isotype control, data not shown), bright CD30, mostly bright CD5 (with a noticeable nonrosetted CD5 low to negative population), bright CD15 at a level slightly lower than granulocytes, and bright CD71 (transferrin receptor) consistent with high metabolic activity. Finally, the population shows a tight cluster in multiple projections including CD95 (bright) versus CD40. In this case, the HRS population represented 0.09% of the total white cells in the lymph node. The case was morphologically confirmed as CHL-nodular sclerosis type.

Further development of a reliable method of diagnosis of classical Hodgkin lymphoma by flow cytometry has benefited from the advances in the instrumentation. Introduction of rapid digital event acquisition on the modern cytometers now allows for the routine analysis of 500,000 events or more within less than 5 minutes, bringing a population that represents 0.01% of the total white cells well within range of sensitivity of clinical cytometry. In addition, increased numbers of lasers and detectors on the modern instruments allow for simultaneous interrogation of 10 or more antigens within the same tube. This, in turn, increases confidence in identification of small populations based on multidimensional analysis of multiple antibody staining patterns on single cells [12]. In our practice, using a nine-antibody combination in a single tube (currently CD5-ECD, CD15-APC, CD20-PE-Cy7, CD30-PE, CD40-PE-Cy5.5, CD45-APC-H7, CD64-FITC, CD71-APC-A700, and CD95-PB) allows for detection of cases with a relatively low abundance of HRS cells even with some deviation from the canonical CD30 and CD15 bright immunophenotype [9, 10]. Such detection relies on stepwise exclusion of reactive populations (CD20-positive B cells, CD5-positive T cells, and CD64-positive monocytes and granulocytes) and refinement of the population of interest with the most common immunophenotype of CD15 (intermediate to bright), CD30 (intermediate to bright), CD40 (bright), CD71 (bright), and CD95 (bright) in multiple projections. Figure 3 demonstrates the usage of this strategy to detect a relatively small malignant population in a CHL lymph node.

In a recent blinded single-center clinical validation study that included common morphologic and immunophenotypic mimics of CHL, the above flow cytometry reagent combination demonstrated an 88.7% sensitivity and 100% specificity for diagnosing CHL. Interestingly, many false negative cases were greater than 72 hours in age, possibly suggesting selective loss of HRS cells during prolonged sample storage [10]. While we have not specifically analyzed the diagnostic sensitivity of different sample types, very paucicellular samples from small biopsies and fine needle aspirations (FNA) may not have similar sensitivity. Our study specifically excluded samples that contained less than 50,000 viable cells due to the expected frequency of HRS cells being lower than the cutoff required for definite abnormal population identification. 

Flow cytometry offers several potential benefits in the diagnosis of CHL. These include: (1) increased diagnostic certainty in cases which may be equivocal or possibly negative by initial morphologic review; our study identified four such cases, which were subsequently confirmed by extensive immunohistochemical panels or tissue section morphology on a subsequent biopsy; (2) rapid turn around time (hours compared to days) required for confirmation of CHL by immunohistochemistry; and (3) significant cost savings compared to extensive immunohistochemical panels that have become common in the morphologic diagnosis of CHL.

There are still several significant limitations of this testing strategy. So far, flow cytometry data has been produced by a single institution and clinical validation at other centers would be necessary before widespread adoption of this method for routine clinical use. Our initial validation of clinical testing for CHL has relied on a 9-color tube. The instruments that are required for such detection are still not widely used in most clinical cytometry laboratories. However, they are commercially available and offer multiple advantages for other clinical applications. We are now in the process of validating a six-color assay that will allow more laboratories to use the methodology. Currently, the detection of HRS cells is limited to tissue samples and has not been validated in either peripheral blood or bone marrow samples. Finally, in cases where the immunophenotype of the cells is consistent with CHL, other related neoplasms must still remain on the differential diagnosis. These include cases of so-called grey zone lymphoma with features intermediate between classical Hodgkin lymphoma and B cell lymphoma as well as other B and T cell lymphomas with HRS-like cells. In the case of grey zone lymphoma, which may present with sheet-like proliferation of HRS-like cells, an important observation is the significantly increased number of malignant cells detected by flow cytometry. In cases of other B and T cell lymphomas, routine flow cytometry panels for B and T cells are usually sufficient to exclude these possibilities. In our practice, we have encountered several such cases following initial validation. These included HRS-like cells in a case of angioimmunoblastic T cell lymphoma, that showed an HRS cell population and aberrant T cell population by flow cytometry and two cases of chronic lymphocytic leukemia/small lymphocytic lymphoma (CLL/SLL) with HRS-like cells that both demonstrated a predominant abnormal CD5+ B cell populations in addition to HRS-cell populations. While in none of the cases did flow cytometry provide a misleading diagnosis, the occurrence of cells with HRS-like phenotype in non-Hodgkin lymphomas further highlights the necessity of morphologic and flow cytometric correlation as a part of complete clinical evaluation.

## 3. Study of Inflammatory Background as an Ancillary Diagnostic Test

A majority of the cellularity in CHL tumors is composed of an inflammatory background. Thus, it is not surprising that a significant amount of effort has been placed in developing diagnostically useful flow cytometric tests focused on evaluation of various subsets of inflammatory cells. T cells have been of particular interest because of the variety of immunophenotypically distinct subsets that can be analyzed. Some, but not all, reports note increased ratios of CD4 to CD8-positive T cells in CHL, compared to reactive lymph nodes by either flow cytometry or immunohistochemistry [13–15]. An increase in T regulatory cells with a CD4, CD25, CD152, and FoxP3 immunophenotype has been identified in multiple studies by either flow cytometry or immunohistochemistry [15–18]. Similar populations were also increased in the blood of many patients with CHL [19]. Additionally, a recent investigation has demonstrated a significantly increased proportion of CD4+ and CD26− T cells in CHL compared to reactive nodes with follicular hyperplasia [20]. These cells possessed both characteristics of T regulatory cells and recently discovered immunomodulatory Th17 cells by cytokine profiling. As T regulatory cells have prominent roles in suppressing cytotoxic immune responses, these findings raise intriguing questions about the cellular milieu that supports the growth of HRS cells and protects them from immune mediated demise. 

**Figure 4:**
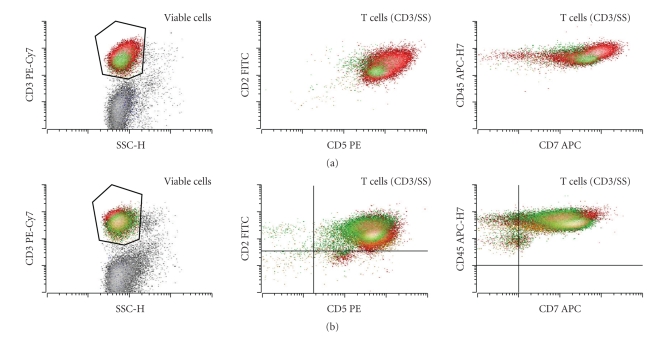
(a) CHL reactive background T cells often demonstrate a range of specific reactive features including increased levels of CD2, CD5, CD7, and CD45 that are particularly noticeable on the CD4-positive T cells (red) compared to CD8-positive T cells (green). Also note increased CD4 : CD8 ratio among the T cells that is often, but not always, observed in CHL cases. (b) No such changes are seen in a patient with reactive follicular hyperplasia.

Additional subsets of CD4- and CD8-positive T cells showing overexpression of CD7 have recently been demonstrated in CHL [21]. Other antigens including CD2, CD5, and CD45 were also increased on the CD4-positive T cells, and CD5 and CD45 were increased on CD8-positive T cells [21, 22]. The increased expression of CD7 and CD45 on the CD4-positive T cells in particular was seen in morphologically involved, but not in the morphologically uninvolved, lymph nodes of the patients with CHL who had multiple biopsies, suggesting a role for the local intra-tumoral milieu on the differentiation of this subset [22].  Figure 4 shows a representative example of a distinct CD4-positive subset with increased CD2, CD3, CD5, CD7, and CD45 in a case of CHL.

The role of this subset and its relationship to Th17 or T regulatory subsets is unclear. While prospective data on the use of these subsets in diagnostic workup of CHL has not been reported, retrospective data showed the sensitivity of approximately 70% and specificity of 90% in the data set assembled by Seegmiller and colleagues [21]. Thus, the identification of either increased numbers of T regulatory cells or CD4-positive T cells with CD7 and CD45 overexpression may serve as a useful adjunct or screening test for CHL in the proper clinical context.

## 4. Flow Cytometric Isolation of HRS Cells

In addition to clinical detection of HRS cells as an adjunct test for CHL diagnosis, flow cytometry offers a significant opportunity to advance research investigations into this poorly understood disease. Until recently, the only applicable methods for cell enrichment were laser capture microdissection of individual HRS cells from tissue sections and magnetic bead-based enrichment using anti-CD30 antibody linked beads. While laser capture microdissection allows for limited molecular analysis, it possesses multiple disadvantages compared to flow cytometric cell sorting including limiting numbers of cells that can be reasonably isolated, unpredictable levels of contamination from underlying reactive cells in the section, and the impossibility of obtaining viable tumor cells. The use of anti-CD30 antibody linked beads has also been described, but the purity of the enriched population from many cases is limiting for even molecular analysis [8]. Flow cytometric sorting, on the other hand, allows for rapid isolation of thousands of viable HRS cells from a single case at purities of 90–95%. Populations of even greater purity, albeit with some loss of yield, can be obtained by two rounds of cell sorting. This technique provides the opportunity to define in vitro growth conditions for HRS cells that may allow for tissue culture-based manipulation. A relatively clean separation of HRS cells from inflammatory background also allows for comparative molecular studies between HRS cells and the inflammatory cells from within the tumor. In one recent example, a loss of heterozygosity of Kelch midbody protein associated with familial CHL was also demonstrated in sporadic CHL using comparison to intratumor T cells [23]. Other applications surely will follow.

## 5. Flow Cytometry for Nodular Lymphocyte Predominant Hodgkin Lymphoma (NLPHL)

While major strides have been made in flow cytometric detection of CHL, relatively little is known about nodular lymphocyte predominant Hodgkin lymphoma. CHL and NLPHL share similar frequency of large malignant cells in an inflammatory background, but unlike CHL, the malignant cells in NLPHL are generally negative for CD30 and CD15 and express the usual B cell-markers CD20 and CD19 as well as normal levels of panhematopoietic marker CD45 [5]. These features make these cells harder to distinguish from nonneoplastic B cells or from neoplastic cells in other B cell lymphomas within an uncharacterized sample. The development of clinically useful testing to directly detect neoplastic cells will require further evaluation of more sensitive and specific markers.

Other clues to the diagnosis may come from analyzing the patterns of the inflammatory cell background that accompanies the tumors. A recent publication demonstrated that detection of significant proportions of CD4 and CD8 double-positive T cells may suggest a diagnosis of NLPHL by flow cytometry [24]. This study showed that many cases of NLPHL show T cells having CD4 expression at normal levels and variable, usually low level of CD8 [24]. Approximately half the cases of NLPHL demonstrated increases in this unusual cell population. These populations were infrequent in CHL and nodular hyperplasia but could be appreciated in a significant proportion of reactive lymph nodes showing progressive transformation of germinal centers (PTGC) and in TCRLBCL. These findings are intriguing as PTGC [5, 25] and TCRLBCL [26] are frequently considered to be related to NLPHL as a possible precursor lesion and a transformation pathway, respectively. Finally, cells with similar immunophenotype have been shown to express a senescence marker CD57 [27] and may correspond to CD57-positive T cells that are commonly increased in NLPHL and sometimes observed rosetting the neoplastic cells [28–30]. Unfortunately, the relatively low sensitivity and specificity of finding a CD4- and CD8-positive T cell population seems to preclude this analysis from being widely useful diagnostically at this time.

## 6. Future Directions and New Findings in Flow Cytometric Analysis of Hodgkin Lymphoma

While HRS cells with their distinctive immunophenotype can now be readily identified and isolated from CHL tumors, the question remains as to whether these cells represent the only malignant component or whether some other, less distinct subset of cells, may serve as a reservoir that gives rise to these cells. This question is not trivial as it may well affect development of targeted therapies for CHL, monitoring of the disease, and our understanding of its pathogenesis. A recent article has demonstrated that in patients with CHL, a rare subset of peripheral blood-based B cells carries the same clonal B cell rearrangement as HRS cells within the tumor [31]. The authors postulated that these CD27+ Aldehyde dehydrogenase-positive B cells serve as the stem cells of Hodgkin lymphoma. A possibly related population of cells that possesses the ability to actively efflux the DNA-binding dye Hoechst 3341 has also been reported [32]. This finding is of importance as such populations are enriched in tissue specific stem cells and may serve as progenitor cells in other neoplasms [33–51]. Further data is certainly required for evaluation of the clinical significance of these very intriguing findings. If these cells do represent CHL precursor cells, their detection in the setting of treatment may be of paramount importance in monitoring clinical response and designing new therapies and may be important for prognosis of the disease. 

We hypothesize that flow cytometry could also be successfully applied to prognostication of clinical outcome of Hodgkin lymphoma. Several recent studies that used either immunohistochemistry or molecular expression array profiling have demonstrated that specific patterns of the inflammatory milieu or antigen expression in HRS cells themselves correlate with prognosis in CHL. However, tissue-based antibody staining studies are very difficult to reproducibly apply in the clinic due to the at best semi-quantitative nature of staining assessment in tissue sections. We believe that most of the markers could be readily portable to a more rapid and quantitative flow cytometry-based method. In particular, expression of Bcl-2 on the HRS cells has been shown to be a marker of poor prognosis [52, 53]. Additional markers such as CD20 expression on HRS cells have also been identified in some studies as a sign of either poor [54, 55] (Blood. 1999; 94:598a) or better prognosis [53, 56]. Both Bcl-2 and CD20 expression can be readily assessed on HRS cells by flow cytometry. 

Similar techniques could be applied for the assessment of the tumor microenvironment-based predictive markers. Expression of markers on various cell subsets within the microenvironment and composition of the inflammatory background have been strongly linked to CHL prognosis [53, 57–62]. A recent publication has used the frequency of CD68-positive macrophages within the tumor milieu to define subgroups with excellent and poor prognosis [63]. These studies suggest that it may be possible to identify patients at greater risk for worse outcome based on expression of antigens by HRS cells themselves or the composition of the tumor milieu. Flow cytometry may well serve as a bridge to bring these exciting research findings into routine clinical practice.
